# Phylogeography, genetic structure and population divergence time of cheetahs in Africa and Asia: evidence for long-term geographic isolates

**DOI:** 10.1111/j.1365-294X.2010.04986.x

**Published:** 2011-02

**Authors:** P Charruau, C Fernandes, P Orozco-terWengel, J Peters, L Hunter, H Ziaie, A Jourabchian, H Jowkar, G Schaller, S Ostrowski, P Vercammen, T Grange, C Schlötterer, A Kotze, E-M Geigl, C Walzer, P A Burger

**Affiliations:** * Department of Biomedical Sciences, Institute of Population Genetics, University of Veterinary MedicineVeterinärplatz 1, 1210 Vienna, Austria; † Research Institute of Wildlife Ecology, University of Veterinary MedicineSavoyenstraße 1, 1160 Vienna, Austria; ‡ Centre for Environmental Biology, Lisbon UniversityCampo Grande, 1749-016 Lisbon, Portugal; § ArchaeoBioCenter and Institute of Palaeoanatomy and History of Veterinary Medicine, Ludwig-Maximilians UniversityKaulbachstrasse 37, 80539 Munich, Germany; ¶ Panthera8 West 40th Street, 18th Fl, New York, NY 10018, USA; ** I.R. Iran Department of Environment, Faculty of Natural Environment and BiodiversityPardisan Park, Hemmat Highway, 11369 Tehran, Iran; †† Conservation of Asiatic Cheetah Project, I.R. Iran Department of EnvironmentPardisan Park, Hemmat Highway, 11369 Tehran, Iran; ‡‡ Wildlife Conservation Society2300 Southern Blvd, Bronx, NY 10460, USA; §§ Breeding Centre for Endangered Arabian WildlifePO Box 29922, Sharjah, United Arab Emirates; ¶¶ Équipe Épigénome et Paléogénome, Institut Jacques Monod, CNRS-Université Paris Diderot15, Hélène Brion, 75013 Paris, France; *** Department of Genetics, University of the Free StateBloemfontein 9300, South Africa; ††† National Zoological Gardens of South AfricaPretoria 0001, South Africa

**Keywords:** *Acinonyx jubatus*, conservation genetics, divergence time, phylogeography, population genetics, subspecies

## Abstract

The cheetah (*Acinonyx jubatus*) has been described as a species with low levels of genetic variation. This has been suggested to be the consequence of a demographic bottleneck 10 000–12 000 years ago (ya) and also led to the assumption that only small genetic differences exist between the described subspecies. However, analysing mitochondrial DNA and microsatellites in cheetah samples from most of the historic range of the species we found relatively deep phylogeographic breaks between some of the investigated populations, and most of the methods assessed divergence time estimates predating the postulated bottleneck. Mitochondrial DNA monophyly and overall levels of genetic differentiation support the distinctiveness of Northern-East African cheetahs (*Acinonyx jubatus soemmeringii*). Moreover, combining archaeozoological and contemporary samples, we show that Asiatic cheetahs (*Acinonyx jubatus venaticus*) are unambiguously separated from African subspecies. Divergence time estimates from mitochondrial and nuclear data place the split between Asiatic and Southern African cheetahs (*Acinonyx jubatus jubatus*) at 32 000–67 000 ya using an average mammalian microsatellite mutation rate and at 4700–44 000 ya employing human microsatellite mutation rates. Cheetahs are vulnerable to extinction globally and critically endangered in their Asiatic range, where the last 70–110 individuals survive only in Iran. We demonstrate that these extant Iranian cheetahs are an autochthonous monophyletic population and the last representatives of the Asiatic subspecies *A. j. venaticus.* We advocate that conservation strategies should consider the uncovered independent evolutionary histories of Asiatic and African cheetahs, as well as among some African subspecies. This would facilitate the dual conservation priorities of maintaining locally adapted ecotypes and genetic diversity.

## Introduction

**Fig. 1:**
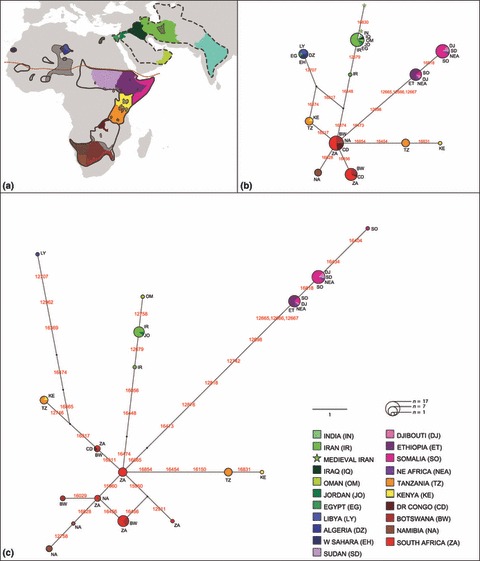
Median-joining (MJ) networks showing phylogeographic structure in African and Asiatic cheetahs. (a) Geographical distribution of the cheetah subspecies and sample repartition. Solid and dashed lines represent the historical distributions of the African and Asiatic cheetah subspecies, respectively (Nowell & Jackson 1996; Krausman & Morales 2005). Hatched fields correspond to current cheetah populations (Durant *et al.* 2008). The different colour shades refer to the screened cheetah subspecies, *Acinonyx jubatus jubatus* (red), *A. j. raineyi* (yellow), *A. j. soemmeringii* (purple), *A. j. venaticus* (green), and to the North African cheetah population (blue). Stars indicate the archaeological sites of Bastam and Takht-e Suleyman, Iran. The dotted line represents the southern boundary of the Sahara. The background map was retrieved from http://www.planiglobe.com (accessed 14 January 2010). (b) MJ-network based on the 139-bp concatenated mitochondrial sequence alignment of 94 samples. (c) MJ network based on the 915-bp concatenated mitochondrial fragment obtained from 62 modern and 16 historical cheetah samples. The consensus networks of all the shortest trees are shown. The specimens included are colour-coded according to their geographical origins (country codes following ISO 3166-Alpha 2). Small black squares represent median vectors, which correspond to either homoplasies or missing haplotypes. Red numbers above lines refer to nucleotide mutations separating the haplotypes [numbering according to GenBank (Accession no. GI:38349475.1)]. Positions 12665–12667 correspond to a 3-bp indel in MT-ND5, which we parsimoniously considered as a single evolutionary event. Exact positions of the concatenated mitochondrial fragments are given in Table S1.

At the end of the nineteenth century, cheetahs were widespread across Africa and much of Asia, ranging from the Indian peninsula to Kazakhstan and Southwest Asia (Pocock 1939; Durant *et al.* 2008). Today only fragmented populations remain on both continents (Durant *et al.* 2008) and are traditionally classified in four African and one Asiatic subspecies (Fig. 1a) (Krausman & Morales 2005). Despite its vast geographical distribution over two continents, the cheetah is regarded as a genetically depauperate species (O’Brien *et al.* 1987; May 1995; Menotti-Raymond & O’Brien 1995; O’Brien & Johnson 2005). This low genetic variability is considered to be the result of a bottleneck at the end of the Pleistocene [10 000–12 000 years ago (ya); O’Brien *et al.* 1987; Menotti-Raymond & O’Brien 1993; O’Brien & Johnson 2005] and has been offered as a possible explanation for the population decline. However, there is little evidence of inbreeding depression in wild cheetahs (Caro & Laurenson 1994). In fact, anthropogenic habitat modification, replacement of wild prey with livestock and concomitant persecution by people (Laurenson 1994; Durant *et al.* 2008; Marker *et al.* 2008) account for the dramatic decline in historical range and numbers (Caro & Laurenson 1994). While it is unclear if Asiatic populations ever reached the density of their African counterparts, historical records report large numbers of cheetahs in Asia until the nineteenth century. During the Middle Ages and early Modern Times, Mughal emperors, in particular Akbar the Great (1556–1605), were known to keep thousands of cheetahs as hunting aids (Pocock 1939; Nowell & Jackson 1996; Allsen 2006; Divyabhanusinh 2007). This practice spread to Europe (Masseti 2009) and Southwest Asia (Brehm 1879; Allsen 2006) until cheetahs became rare, which led to regular imports of individuals from East Africa (Pocock 1939; Divyabhanusinh 2007) into India during the European colonial era. Until now, only sub-Saharan populations (Menotti-Raymond & O’Brien 1995; Freeman *et al.* 2001; Burger *et al.* 2004; Kotze *et al.* 2008; Marker *et al.* 2008) and a few Algerian individuals (Busby *et al.* 2009) have been investigated using genetic markers. Accordingly, comprehensive data regarding the relationships among all African subspecies and between African and Asiatic cheetah populations are still lacking.

In this study, we investigated the phylogeography, genetic structure and evolutionary history of cheetahs from most extant and recently extinct populations in Africa and Asia. We give particular attention to the Asiatic cheetah, because it is critically endangered and restricted to a small remnant population in Iran and possibly a few individuals in Pakistan (Farhadinia 2004) and Afghanistan (Manati & Nogge 2008). Asiatic cheetahs are known to occur in 13 sites in central and northern Iran where the total population is estimated at 70–110 (Farhadinia 2004; CACP 2008). Widespread poaching of the cheetah’s prey base and persecution by local livestock herders are the main causes of the cheetah’s recent decline and, together with road accidents, are likely the limiting factors to their recovery today (Hunter *et al.* 2007; CACP 2008). Historical records of extinction in the Arabian Peninsula indicate that this population became progressively and ultimately isolated from any potential link to Africa between approximately 1950 and 1980 (Hunter & Hamman 2003). However, it was unclear if demographic and genetic exchange between African and Asiatic cheetahs occurred prior to this recent anthropogenic isolation. To investigate these questions, we apply palaeogenetic analyses to compare extinct and extant Asiatic cheetahs with the major African populations. By demonstrating that all Northern-East African individuals, as well as all Asiatic cheetahs group within independent clusters, clearly distinct from other genotypes and monophyletic for mitochondrial DNA (mtDNA), we identify these two populations as long-term geographic isolates. The identification of taxonomic and populations units, and understanding their evolutionary relationships, is essential for the conservation of biological diversity (Allendorf & Luikart 2007). Within species, preservation of genetically distinct local populations maintains evolutionary processes and potential and minimizes extinction risks (Frankham *et al.* 2009).

## Materials and methods

### Sample collection

Details on the origin and sample type of the 94 cheetahs included in this study are provided in Table 1. Modern samples were either collected non-invasively, during routine veterinary treatment, or post mortem. The osseous remains of cheetahs analysed in this study were collected from the archaeological sites of Bastam and Tahkt-e Suleyman in the Province of West Azerbaijan, Northwest Iran. Bastam is situated ∼50 km north of the city of Khvoy, close to the Turkish border at an altitude of 1300 m. First inhabited in Urartian times and destroyed by a fire *c*. 650 bce (Before Common Era), Bastam was reoccupied during the Median and Persian period (550–330 bce) and finally in mediaeval times. Archaeological excavations in the 1970s produced a large faunal assemblage (*n* = 26 987) (Boessneck & Krauß 1973; Krauß 1975). From a mediaeval context (9th–15th century ce), a complete *Os metatarsale IV* was recovered. Originally described as a wolf metatarsal (Boessneck & Krauß 1973), the specimen was re-identified after a thorough comparison with fourth metatarsal bones of modern *Acinonyx jubatus*. The site of Tahkt-e Suleyman (lit. ‘Throne of Salomon’) lies midway between Urmia and Hamadan, about 30 km north of the town of Takab at an altitude of *∼*2000 m. Archaeological excavations carried out in the 1960s revealed Achaemenid/Persian, Parthian, Sassanid and mediaeval inhabitation, and produced a faunal assemblage of *∼*4000 bones. Kolb (1972) assigned a mandible, a cervical vertebra, and left and right coxal bones to *A. jubatus*, based on morphological criteria. The presence of postcranial fragments is strongly indicative of a local origin of the animal(s) and not of an individual whose pelt had been traded into the site. The mandible, vertebra and one coxa from the site of Tahkt-e Suleyman, as well as the metatarsus from Bastam, were subjected to palaeogenetic analysis. All cheetah bones date to the 9th and/or 10th centuries ce.

**Table 1:** Details on the samples used in this study

No.	ID	Type	Origin (time*)	Collection place
1	AJI 01	Faeces	Iran (IR)	IR
2	AJI 02	Faeces	IR	IR
3	AJI 03	Faeces	IR	IR
4	AJI M1A	Faeces	IR	IR
5	AJI M2A	Faeces	IR	IR
6	AJI T	Faeces	IR	IR
7	AJI 08	Faeces	IR	IR
8	AJI 11	Faeces	IR	IR
9	AJI 04	Museum tissue	IR	Sharjah, AE
10	HZM 2.26502	Hide	Oman (OM; 1977)	HZM, GB
11	ZMB 56122	Hide	Jordan (JO)	NHM Berlin, DE
12	BMNH ZD 1939.536	Hide	IR	NHM London, GB
13	SAPM-Gepard-1a-F6-Ba	Metatarsal bone	IR, Bastam (800–900 ce)	SAP Munich, DE
14	SAPM-Gepard-1b-TeS1	Mandible + vertebra	IR, Tahkt-e Suleyman (800–900 ce)	SAP Munich, DE
15	BMNH ZD 1943.56	Museum tissue	Iraq (IQ; 1928)	NHM London, GB
16	BMNH 32.4.7.1	Hide	India (IN; 1925)	NHM London, GB
17	SMNS 18941	Maxillot. bone	Egypt (EG; T. v.Heuglin; 1850s)	SMN Stuttgart, DE
18	SMF 58993	Hide	EG, Libyan Desert (1974)	SM Frankfurt, DE
19	NMW 12071	Hide	Libya (LY)	NHM Vienna, AT
20	NMW 12070	Hide	LY	NHM Vienna, AT
21	BMNH ZD 1957.312	Hide	LY (1955)	NHM London, GB
22	BMNH ZD 1939.1685	Museum tissue	Algeria (DZ)	NHM London, GB
23	ZMB 56277	Maxillot. bone	Western Sahara (EH)	NHM Berlin, DE
24	ZMB 42242	Hide	EH, Rio d’Oro	NHM Berlin, DE
25	ADJ 2	Hair	Ethiopia (ET), custom’s seizure	DECAN, DJ
26	ADJ 3	Hair	ET, custom’s seizure	DECAN, DJ
27	ADJ 4	Hair	ET, custom’s seizure	DECAN, DJ
28	ADJ 5	Hair	ET, custom’s seizure	DECAN, DJ
29	ADJ 6	Hair	ET, custom’s seizure	DECAN, DJ
30	ADJ 8	Hair	Djibouti (DJ)	DECAN, DJ
31	#4421	Skin	Somalia (SO)	Sharjah, AE
32	#4499	Skin	SO	Sharjah, AE
33	#4500	Skin	SO	Sharjah, AE
34	#4203	Skin	SO	Sharjah, AE
35	#4208	Skin	SO	Sharjah, AE
36	#4202	Skin	SO	Sharjah, AE
37	#4223	Skin	SO	Sharjah, AE
38	#4205	Skin	SO	Sharjah, AE
39	#4229	Skin	SO	Sharjah, AE
40	#4228	Skin	SO	Sharjah, AE
41	#4206	Skin	SO	Sharjah, AE
42	#4201	Skin	SO	Sharjah, AE
43	#4418	Skin	SO	Sharjah, AE
44	#4222	Blood	SO	Sharjah, AE
45	#4216	Skin	SD	Sharjah, AE
46	LP4304	Hair	Northern-east Africa (N-E.A)	La Palmyre, FR
47	#4415	Skin	N-E.A	Sharjah, AE
48	SMNS 38432	Hide	DJ, custom’s seizure (<1985)	SMN Stuttgart, DE
49	ADJ 1	Hair	ET, custom’s seizure	DECAN, DJ
50	ADJ 7	Hair	ET, custom’s seizure	DECAN, DJ
51	Claudia	Faeces	Kenya (KE)	DECAN, DJ
52	ZMB34306	Maxillot. bone	Tanzania (TZ)	MHN Berlin, DE
53	ZMB56287	Maxillot. bone	TZ	MHN Berlin, DE
54	ZMB56302	Maxillot. bone	TZ	MHN Berlin, DE
55	ZMB56306	Maxillot. bone	TZ	MHN Berlin, DE
56	ZMB56309	Maxillot. bone	TZ	MHN Berlin, DE
57	Tigger	Faeces	KE	DECAN, DJ
58	ZMB56128	Maxillot. bone	TZ	MHN Berlin, DE
59	ZMB56289	Maxillot. bone	TZ	MHN Berlin, DE
60	ZMB56293	Maxillot. bone	TZ	MHN Berlin, DE
61	ZMB56299	Maxillot. bone	TZ	MHN Berlin, DE
62	GACH 18/08	Blood	South Africa (ZA)	Pretoria NZG, ZA
63	GACH 23/06	Blood	ZA	Pretoria NZG, ZA
64	GACH 26/08	Blood	ZA	Pretoria NZG, ZA
65	GACH 33/08	Blood	ZA	Pretoria NZG, ZA
66	GACH 35/08	Blood	ZA	Pretoria NZG, ZA
67	GACH 38/08	Blood	ZA	Pretoria NZG, ZA
68	GACH 42/08	Blood	ZA	Pretoria NZG, ZA
69	GACH 44/08	Blood	ZA	Pretoria NZG, ZA
70	GACH 45/08	Blood	ZA	Pretoria NZG, ZA
71	#1463	Lung	Namibia (NA) descendant	La Palmyre, FR
72	#1557/NMSZ 2001.37	Muscle	NA	NMSZ, GB
73	S1571	Muscle	ZA descendant	Private owner, DE
74	1921	Muscle	NA descendant	Zoo Salzburg, AT
75	#3155/NMSZ 2000.151.2	Muscle	NA descendant	NMSZ, GB
76	#3240	Faeces	ZA descendant	Zoo Vienna, AT
77	GACH 34/08	Blood	ZA	Pretoria NZG, ZA
78	#3779/NMSZ 1999.221	Muscle	ZA descendant	NMSZ, GB
79	Douma	Muscle	ZA	Zoo Lunaret, FR
80	GACH 25/08	Blood	ZA	Pretoria NZG, ZA
81	GACH33	Blood	ZA	Pretoria NZG, ZA
82	GACH 01/08	Blood	Botswana (BW)	Pretoria NZG, ZA
83	GACH 02/08	Blood	BW	Pretoria NZG, ZA
84	GACH 11/08	Blood	BW	Pretoria NZG, ZA
85	GACH 12/08	Blood	BW	Pretoria NZG, ZA
86	GACH 15/08	Blood	BW	Pretoria NZG, ZA
87	GACH 16/08	Blood	BW	Pretoria NZG, ZA
88	#4268	Skin	NA	Sharjah, AE
89	#2486/ZFMK 2005.357	Hide	ZA, king cheetah	MHN Bonn, DE
90	RMCA 454	Maxillot. bone	D.R.Congo (CD)	RMCA, BE
91	RMCA 1236	Maxillot. bone	CD	RMCA, BE
92	RMCA 19237	Maxillot. bone	CD	RMCA, BE
93	RMCA 22347	Maxillot. bone	CD	RMCA, BE
94	RMCA 22390	Maxillot. bone	CD	RMCA, BE
95	*Puma concolor*	Blood	Unknown	Zoo Salzburg, AT

* Date of collection of historical samples, if available.

#: registration number in the international cheetah studbook (Marker 2009); maxillot. bone: maxilloturbinate bone; museum tissue: dried tissue remaining on the skull; IR: Ariz & Bafq Protected Area, eastern Yazd province, Naybandan Wildlife Refuge south of Tabas, IR; Sarjah: Breeding Centre for Endangered Arabian Wildlife, Sharjah, AE; HZM: Harrison Institute, Sevenoaks, GB; NHM: Natural History Museum; SAP: State Collection of Anthropology and Palaeoanatomy, Munich, DE; SMN: Museum of Natural Sciences, Stuttgart, DE; SMN: Naturmuseum Senckenberg, Frankfurt, DE; DECAN: DECAN rescue centre, Djibouti, DJ; La Palmyre: Parc zoologique de La Palmyre, FR; Zoo Lunaret: Parc Zoologique Henri de Lunaret, Montpellier, FR; NZG: National Zoological Gardens, Pretoria, ZA; NMSZ: National museum of Scotland, Edinburgh, GB; RMCA: Royal Museum of Central Africa, Tervuren, BE. Country codes following ISO 3166-Alpha 2.

### DNA extraction

Ancient DNA extractions from mediaeval cheetah specimens were performed in highly contained laboratories of the palaeogenetic core facility at the Institute Jacques Monod, Paris (see Supporting Information). The superficial layer of the small bone fragments was removed and samples were ground to a fine powder in a freezer mill (Freezer Mill-6750; Spex Certiprep). The bone powder (*∼*180 mg) was incubated (37 °C; 48 h) in extraction buffer (0.5 m EDTA pH 8.0; 0.25 m sodium phosphate buffer pH 8.0; 1 mmß-Mercapto-ethanol). The extract was purified according to an improved protocol using the QIAquick gel extraction kit (Qiagen). Museum skin pieces were incubated twice (24 h) in TE buffer to remove potential enzyme inhibitors (Johnson *et al.* 2004). Complete enzyme digestion was carried out in an improved lysis buffer [100 mm Tris–HCl pH 8.0; 100 mm NaCl; 3 mm CaCl_2_; 2%*N*-lauroyl-sarcosyl (NLS); 40 mm DTT; 5 mm PTB (*N*-phenacyl-thiazolium-bromide (Vasan *et al.* 1996) in 10 mm phosphate buffer); 340 μg proteinase K] (Pfeiffer *et al.* 2004). After 24 h one-eighths of an Inhibitex pill (Qiagen) was added to the samples, which had extensively undergone a tanning process. Maxilloturbinate bone shreds (Wisely *et al.* 2004) were ground and the bone powder was incubated (56 °C; 48 h) in lysis buffer (0.5 m EDTA pH 8.0; 0.25 m sodium phosphate buffer pH 8.0; 1 mmß-Mercapto-ethanol; 2% NLS; 340 μg proteinase K). DNA extraction was performed with commercial kits (Qiagen) in the presence of negative controls. Genomic DNA of modern samples was isolated from blood and tissue with the NucleoSpin®-Tissue Kit (Macherey-Nagel). Faeces were processed, following a two-step storage protocol (Nsubuga *et al.* 2004), with the QIAamp DNA Stool Mini Kit (Qiagen). Hair samples were digested with a lysis buffer (Pfeiffer *et al.* 2004) and DNA was extracted with the NucleoSpin®-Tissue Kit (Macherey-Nagel). Two independent extractions were carried out for the mediaeval bones, as well as for the other samples where sufficient material (i.e. museum specimen) was available.

### Quantitative real-time polymerase chain reaction and sequencing of mediaeval cheetah specimens

Fragments of 139 base pairs (bp), including 14 informative polymorphisms in NADH-dehydrogenase subunit 5 (MT-ND5) and control region (MT-CR) (Tables 2 and S1), were amplified from two mediaeval and 14 museum specimens by UNG-coupled quantitative real-time polymerase chain reaction (UQPCR) (Pruvost *et al.* 2005) with the LightCycler® FastStart DNA MasterPLUS SYBR Green I mix (Roche Diagnostics GmbH). Quantification of initial target molecules was performed using a titration curve established with a homologous reference (DNA from a Namibian specimen of *A. j. jubatus*) according to Pruvost *et al.* (2005, 2007). The inhibition of the polymerase by the aDNA extracts was quantified (Pruvost & Geigl 2004) and the applied amount of target DNA was adjusted accordingly. The characterization of the PCR products was performed by analysis of the fusion temperature (*T*_m_) using the Lightcycler® and via electrophoresis in a 10% polyacrylamide gel. Multiple PCR amplifications were performed on each independent extract. PCR products were purified using the QIAquick PCR purification kit (Qiagen) and sequenced in both directions by Eurofins MWG GmbH.

**Table 2:** Informative sites screened in the 139-bp mtDNA concatenated fragment

Amplicons	nt	*A. j.* *venaticus*	*A. j.* *jubatus*	*A. j.* *soemmeringii*	North Africa population	*A. j.* *raineyi*
MT-ND5	12665–12667	ATC	ATC	—	ATC	ATC
	12679	T/C	C	C	C	C
	12698	C	C	**A**	C	C
	12707	A	A	A	**G**	A
MT-CR1	16448	**T**	C	C	C	C
	16454	T	T	T	T	C/T
	16473	T	T	**C**	T	T
	16474	A	G	G	A	G
MT-CR3	16817	T	T	T	C	C/T
	16818	A	A	G/A	A	A
	16831	A	A	A	A	G/A
	16854	A	A	A	A	G/A

nt: nucleotide position (GenBank Accession no. GI:38349475.1); MT-ND5: mitochondrial NADH-dehydrogenase subunit 5; MT-CR: mitochondrial control region. Diagnostic nucleotide polymorphisms are highlighted in bold. *A. j. venaticus* (*Acinonyx jubatus venaticus*) refers to the Southwest Asian cheetah population.

### DNA sequencing and genotyping

A total of 62 modern and 16 historical cheetah PCR products were sequenced for parts of MT-ND5 [nt 12657–13087; numbering according to GenBank (Accession no. GI:38349475.1)], cytochrome *b* (MT-CB; nt 15940–16173) and MT-CR without repetitive sequences (Lopez *et al.* 1996) (nt 16333–16487 and nt 16811–16876), resulting in a 915-bp concatenated mitochondrial fragment. The last 29 bp of the tRNA Leucine (tRNA-Leu; nt 12628–12656) were analysed to ensure the correct amplification of a 3-bp indel mutation at the third and fourth codons of MT-ND5. Sequencing was performed in both directions using a MegaBACE 500 sequencer (GE Healthcare). Genotypes were obtained from 60 modern and seven historical samples at 20 microsatellite loci (FCA005, FCA008, FCA014, FCA026, FCA069, FCA078, FCA085, FCA096, FCA097, FCA105, FCA126, FCA133, FCA171, FCA212, FCA214, FCA220, FCA224, FCA230, FCA247, FCA310) developed in *Felis catus* (Menotti-Raymond *et al.* 1999) and tested on cheetahs (Driscoll *et al.* 2002; Kotze *et al.* 2008; Marker *et al.* 2008). Southern African sample analyses were performed at the Centre for Conservation Science of the National Zoological Gardens in Pretoria using an ABI 3130 sequencer (Applied Biosystems Inc.). The genotypes of all other samples were determined with a MegaBACE 500 at the University of Veterinary Medicine, Vienna. At least three independent genotype results were produced for each locus and each individual. Two defined standard individuals were run in each genotyping series. Electropherograms were evaluated using the softwares GeneMapper v3.1 (Applied Biosystems) and MegaBACE Genetic Profiler v2.2 (GE Healthcare), respectively. Results from the loci FCA078 and FCA096 were removed because of the insufficient quality of the electropherograms despite multiple reiterations.

### Mitochondrial and nuclear DNA data analysis

Mitochondrial sequences were deposited in GenBank (Accession nos puma, GU984641; cheetah, GU984642–GU984735). Sequences were aligned with Codon Code Aligner (version 3.0.2; Codon Code Corporation). A new polymorphism was considered as authentic when it was displayed in at least three independent sequences. The 3-bp deletion (nt 12665–12667) was considered as a single indel event in all subsequent analysis. Mitochondrial haplotype diversity (*H*_d_) and nucleotide diversity (π) (Tajima 1983; Nei 1987) were calculated using Arlequin 3.5 (Excoffier & Lischer 2010). The genetic structure of cheetah populations was analysed using a Bayesian approach implemented in baps 5.2 (Corander & Marttinen 2006; Corander *et al.* 2008). In this method, the number of populations is treated as unknown parameter and is directly inferred from the data set without defining a prior estimate. For inferring population structure in the mitochondrial (139 bp, *n* = 94; 915 bp, *n* = 60) and nuclear (18 loci, *n* = 60) data sets, we assigned individuals to distinct clusters using the models ‘clustering of linked loci’ (Corander & Tang 2007) and ‘clustering of individuals’, respectively. We specified prior upper bound values for the number of clusters in the data (i.e. 5–10) and performed 10 independent runs for each value. In all independent runs, the assignments of individuals resulted in the same clusters. We performed the admixture analysis based on the results of the mixture clustering of the nuclear data using 500 iterations and a number of 1000 reference individuals per population, each with 10 reiterations. For additional population structure analysis, we used the three-dimensional factorial correspondence analysis (FCA) in genetix 4.05 (Belkir *et al.* 1999), which portrays the relationship between individuals or populations based on the detection of the best linear combination of allele frequencies. By comparing the clustering solutions of the different methods, we defined the cheetah populations for subsequent population genetic analyses. Average polymorphisms, allele frequencies, expected heterozygosities (*H*_E_), pairwise *F*_ST_, genetic distance (δμ)^2^ (Goldstein *et al.* 1995) and proportion of shared alleles between individuals (*D*_PS_; Bowcock *et al.* 1994) were estimated from the microsatellite data set with MSAnalyzer 4.05 (Dieringer & Schlötterer 2003). The stepwise weighted genetic distance (*D*_SW_; Shriver *et al.* 1995) was calculated with Populations 1.2.30 (Langella 1999). Statistical significance for mean *H*_E_ was tested with the Wilcoxon rank-sum test using r version 2.10.1 (R Development Core Team 2009). An analysis of molecular variance (amova) was performed to determine the proportion of genetic variance explained by the differences within and between modern populations as determined by BAPS and FCA. amova calculations were performed in Arlequin 3.5 and significance levels were obtained with 10 000 permutations. Neighbor-joining (NJ) trees were generated based on the proportion of shared alleles between individuals with the software phylip 3.69 (Felsenstein 1989), visualized in FigTree v1.3.1. (Rambaut 2009) and edited in Adobe® Illustrator® CS4 14.0.0 (Adobe System Inc.). We also tested the populations defined by BAPS and FCA for evidence of a decline in their effective population sizes using the program Bottleneck 1.2.0.2 (Piry *et al.* 1999). We performed the evaluation using the stepwise mutation (SMM) and two-phase (TPM) models of microsatellite evolution. The significance of the tests was assessed using Wilcoxon sign-rank test, which is the most appropriate test when fewer than 20 microsatellite loci are used (Piry *et al.* 1999). Median-joining networks were constructed with Network 4.5 (Bandelt *et al.* 1999) with adapted settings following the software instructions. We applied a transition/transversion ratio of 14 (MJN 139 bp) and 10 (MJN 915 bp) estimated with ModelTest (Posada & Crandall 1998) using the best fitting model [Hasegawa–Kishino–Yano (HKY); Hasegawa *et al.* 1985] according to the Akaike Information Criterion (AIC; Akaike 1974). An exact test of population differentiation (Raymond & Rousset 1995) was performed, and the population pairwise *F*_ST_ values were calculated based on the 139- and 915-bp mitochondrial fragment using the distance method of Tamura & Nei (1993) implemented in Arlequin 3.5.

### Divergence time estimations

In all divergence time estimations, we used the most complete sample set (*n* = 67) for which both mitochondrial (915 bp) and nuclear (18 loci) data could be retrieved. The 3-bp deletion was considered as one evolutionary event. A puma sample (*Puma concolor*) was sequenced and included as outgroup to build a maximum-likelihood (ML) tree using the HKY model in Tree-Puzzle 5.2 (Schmidt *et al.* 2003). We tested whether the assumption of a molecular clock was valid by performing a likelihood ratio test between the simpler clock model vs. the more complex model without clock. The log-likelihood of the more complex model was not significantly increased with respect to the simpler model (*P* > 0.05), supporting the assumption of a molecular clock (Felsenstein 1985). Given an estimated cheetah–puma divergence at 4.92 Ma (95% CI = 3.86–6.92) (Johnson *et al.* 2006), the substitution rate was inferred using the formula *d*_*xy*_ = 2μT, where *T* is the time to the most recent common ancestor, μ is the mutation rate per year and *d*_*xy*_ is the genetic distance between species corrected for ancestral polymorphism (Nei 1987). For the computation of *d*_*xy*_, we used the software mega 4.0 (Tamura *et al.* 2007), which allows for rate heterogeneity among lineages, and the Tamura–Nei substitution model (Tamura & Nei 1993) with Γ = 0.118 (parameter selected by the AIC with correction for small sample size; AICc) as selected by Treefinder (Jobb *et al.* 2004). The estimated *d*_*xy*_ was 0.567 (SD ± 0.175), which translates into a substitution rate of 5.76 × 10^−8^ substitutions per site per year (95% CI = 1.57 × 10^−8^–1.19 × 10^−7^). The divergence times between Asiatic (*A. j. venaticus*) and Southern African (*A. j. jubatus*) cheetahs and between Northern-East (*A. j. soemmeringii*) and Southern African cheetahs were estimated using the equation *D*_A_ *=* *2*μ*T* in which μ is the average substitution rate per nucleotide, *T* is the divergence time, and *D*_A_ is the net number of nucleotide differences between populations (Nei & Li 1979). Divergence times were also calculated following the coalescent method described by Gaggiotti & Excoffier (2000). This method aims to remove the effect of bottlenecks and unequal sizes of the derived populations, which can lead to the overestimation of divergence times from genetic distances. Additionally, these divergence times were estimated using IMa (Hey & Nielsen 2007). This program implements a coalescent-based isolation with migration model that can be applied to genetic data drawn from a pair of closely related populations or species (Nielsen & Wakeley 2001) to infer six demographic parameters [population sizes of the extant as well as the ancestor population, migration rates (*m*1, *m*2) per gene in both directions, and time (*t*) since divergence]. After preliminary runs to optimize settings, four replicate simulations were run. Estimates were generated under the HKY model. Simulations used 10 Markov chains, with 45 chain swap attempts per step, and were run for 20 million steps discarding the first 1 million steps as ‘burn-in’. Genealogies were sampled every 100 steps. Convergence of the simulations was assessed by comparison of their marginal parameter distributions across independent replicate runs. Saved genealogies were used to estimate the joint marginal distribution of *t* (the estimator of population divergence time) from an evenly spaced sample of 200 000 trees. To convert coalescent times to years before present, we used the substitution rate estimated above and a generation time of 6 years (Marker & O’Brien 1989). ‘Nested models’ (Hey & Nielsen 2007) were also examined and compared to the full six-parameter model using log-likelihood ratio tests. For comparison with previous divergence time estimates between cheetah subspecies based on microsatellite data (Driscoll *et al.* 2002), we estimated the timing of the splits between Asiatic and African subspecies (*A. j. venaticus* and *A. j. jubatus*) and among African cheetahs (*A. j. soemmeringii* and *A. j. jubatus*) using the (δμ)^2^ genetic distance and the equation (δμ)^2^ = 2μG (μ = mutation rate; *G* = generations) (Goldstein & Pollock 1997). We applied two estimates of mutation rate for microsatellite loci in humans (5.6 × 10^−4^ and 2.05 × 10^−3^), which were previously used by Driscoll *et al.* (2002), and an additional estimate for the average microsatellite mutation rate in mammals (2.05 × 10^−4^; Rooney *et al.* 1999) that has been employed in several studies on other felid species (Spong *et al.* 2000; Anderson *et al.* 2004; Ruiz-Garcia *et al.* 2006). Furthermore, to estimate the divergence times among African populations and between Asiatic and African cheetahs, we used the stepwise-weighted genetic distance (*D*_SW_; Shriver *et al.* 1995) and the equation from Calabrese *et al.* (2001): *D*_SW_ = √(2/π) × √(2βτ + 4β*N*_e_) − 4β*N*_e_/√(8β*N*_e_ + 1), where π is a constant; β, mutation rate; τ, time in generations; *N*_e_, effective population size calculated under the SMM and inferred from the expected heterozygosity (Ohta & Kimura 1973).

## Results

### Genetic variation and population structure analysis

We screened 94 Asiatic and African cheetahs by combining data from 62 modern, 30 historical and 2 zooarchaeological specimens (Table 1). Three mtDNA regions (MT-ND5, MT-CB, MT-CR; partial sequences), corresponding to a total of 915 bp were sequenced from all modern and 16 well-preserved historical specimens. We identified 29 polymorphic sites and one 3-bp deletion resulting in 18 haplotypes (*H*_d_ = 0.909, SD = 0.013; π = 0.00659, SD = 0.00351). The highest numbers of polymorphic sites (*n* = 7) were detected within cheetahs originating from Southern Africa and East Africa, respectively, whereas Northern-East African and Asiatic cheetahs showed lower amounts of mitochondrial polymorphism (*n* = 3 and *n* = 2, respectively). A similar pattern was observed for haplotype (*H*_d_) and nucleotide diversities (π) (Table 3). For the palaeogenetic analyses, we selected three diagnostic regions (139 bp) containing 14 informative sites (Table 2). These sites faithfully recovered the partitioning into haplogroups observed in the 915-bp data set. We successfully amplified these regions in mediaeval *A. jubatus* specimens from two archaeological sites in Iran, Bastam (metatarsal bone) and Tahkt-e Suleyman (vertebra, mandible). These samples represent the few cheetah bones hitherto discovered in archaeological excavations in Southwest Asia. In addition, we amplified these diagnostic regions in 14 highly degraded DNA samples originating from countries where cheetahs are now extinct (e.g. India) or close to extinction. All replicates of the sequences retrieved from the independent extracts were identical.

**Table 3:** Genetic variation in cheetahs inferred from mitochondrial DNA (mtDNA) and nuclear DNA (μsat) data

	mtDNA (915 bp)				
	
Population	No. cheetahs (mtDNA/μsat*)	No. haplotypes	No. variable sites	Haplotype diversity (SE)	π (SE)
Total	78/60	18	29 + 1 indel	0.909 (0.013)	0.00659 (0.00352)
S-West Asia	11/8	3	2	0.345 (0.172)	0.00040 (0.00047)
N-East Africa	26/25	3	3	0.551 (0.048)	0.00073 (0.00064)
Southern Africa	29/27	8	7	0.828 (0.046)	0.00197 (0.00130)
East Africa	11/0	3	7	0.636 (0.090)	0.00381 (0.00237)
North Africa	1/0	1	0	—	—
	μsat (18 loci)				

	% Polymorphic loci	Total no. alleles	Average no. allele/locus (SE)	% Private alleles	*H*_E_
	100	145	8.06 (1.39)	31.72	0.766
	88.9	42	2.33 (1.03)	7.14	0.397
	100	107	5.94 (1.70)	14.95	0.674
	100	111	6.17 (1.38)	24.32	0.698
	—	—	—	—	—
	—	—	—	—	—

* Nuclear genetic variation was assessed only among the extant populations.

**Fig. 2:**
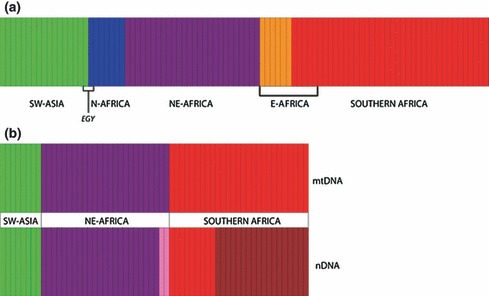
Bayesian analysis of population structure (BAPS) of African and Asiatic cheetahs. (a) Clustering based on a 139-bp mitochondrial concatenated fragment of 94 cheetahs. Individuals (represented by single bars) are assigned to five distinct clusters (posterior probability, PP = 1). (b) Clustering based on a 915-bp mitochondrial fragment and 18 microsatellite loci using 60 modern cheetahs. Extant cheetahs are assigned to three (PP = 0.999) and five (admixture analysis; PP = 0.999) clusters using mitochondrial (mtDNA) and nuclear DNA (nDNA), respectively. SW-ASIA, Southwest Asia; N-AFRICA, North Africa; EGY, Egypt; NE-AFRICA, Northern-East Africa; E-AFRICA, East Africa; S-AFRICA, Southern Africa.

We used haplotype network analysis and BAPS to infer the relationships between the different mtDNA haplotypes. In both median-joining networks (Fig. 1b: 139 bp and Fig. 1c: 915 bp), we observed a star-shaped radiation stemming from a Southern African haplogroup corresponding to the subspecies *A. j. jubatus*. The East African cheetahs, described as *A. j. raineyi*, emerged in two different branches from the central haplotype. Although none of the East African cheetahs shared a common haplotype with the Southern African individuals, one haplotype comprising Tanzanian and Kenyan cheetahs (defined by nt 16817; Fig. 1b) clustered together with Southern African cheetahs in the BAPS analysis [posterior probability (PP) = 1; Fig. 2a]. Another sub-Saharan cheetah haplogroup was defined (Fig 2a) corresponding to the Northern-East African subspecies *A. j. soemmeringii*. We observed a monophyletic clustering for this haplogroup in the ML tree (915 bp) with a bootstrap support of 99% (1000 iterations; Fig. S1). Two more haplogroups were recovered (Fig. 1b) in samples from different parts of a range (North Africa and Southwest Asia) that had previously been considered to harbour the same subspecies *A. j. venaticus* (Krausman & Morales 2005; Belbachir 2007). The partitioning of these cheetahs into two distinct clusters was also confirmed by BAPS (Fig. 2a). One of these clusters encompassed animals from Western Sahara, Algeria, Libya and western Egypt (Libyan Desert; Osborn & Helmy 1980). The other cluster contained three Asiatic haplotypes represented by the current, historic and mediaeval Iranian cheetah samples as well as by museum specimens from India, Oman, Iraq and Jordan (Fig. 1b). In addition, one sample collected by Theodor von Heuglin in eastern Egypt (Table 1) clustered with this Asiatic haplogroup (Figs 1b and 2a). An exact test of population differentiation based on haplotype frequencies resulted in significant differences (*P* < 0.05) between all African and Asiatic clusters as defined by the Bayesian structure analysis (Fig. 2a). The population pairwise genetic distances (*F*_ST_) among these clusters ranged from 0.724 to 0.930 (within Africa) and 0.818–0.958 (Southwest Asia vs. African populations; Table 4). Comparing the *F*_ST_ values among the African clusters with those calculated between Asiatic and African populations, no significant differences were detected (*P* = 0.246; Wilcoxon rank-sum test corrected for multiple testing).

**Table 4:** Population pairwise distances

	S-West Asia	N-East Africa	Southern Africa	East Africa	North Africa
S-West Asia	—	0.295	0.305	na	na
N-East Africa	0.930/0.947	—	0.170	na	na
Southern Africa	0.818/0.689	0.772/0.806	—	na	na
East Africa	0.951/0.951	0.901/0.939	0.724/0.613	—	na
North Africa	0.958/na	0.930/na	0.796/na	0.972/na	—

Population pairwise distances based on the concatenated mitochondrial sequence (below the diagonal: *F*_ST_; 139 bp; *n* = 94/915 bp; *n* = 78) and 18 polymorphic microsatellite loci (above the diagonal*: F*_ST_; *n* = 60). All *F*_ST_*P*-values are significant (*P* < 0.0001). na, not applicable. Populations were defined according BAPS and FCA clustering.

**Fig. 3:**
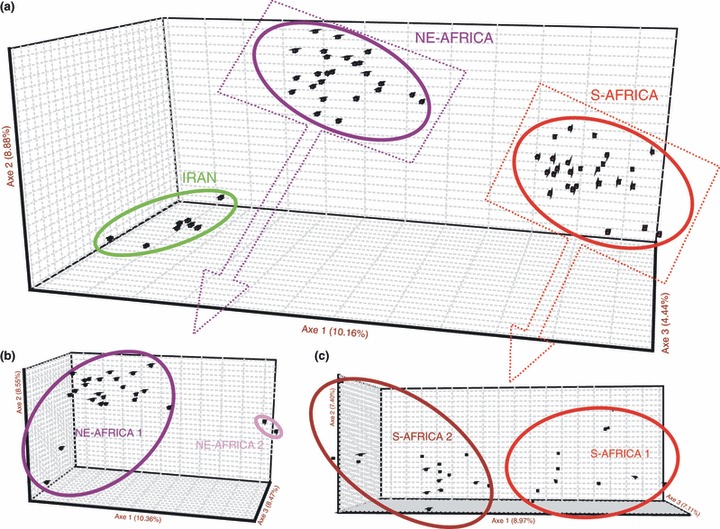
Three-dimensional factorial correspondence analyses (FCA) of African and Asiatic cheetahs based on 18 microsatellite loci. (a) The population structuring of 60 individuals in three clusters corresponding to their geographical origin is shown. The axes 1–4 explain 27.4% of the variation among the populations. (b, c) FCA graphs considering independently the Southern (*n* = 27) and Northern-East African (*n* = 25) cheetah populations. The subclustering within each population reflects the clusters defined with BAPS (Fig. 2b).

**Fig. 4:**
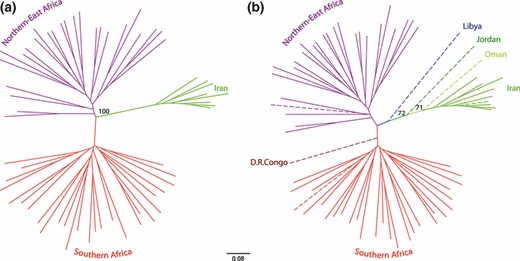
Neighbor-joining (NJ) trees displaying African and Asiatic cheetahs in independent branches. The NJ trees are based on the proportion of shared alleles (*D*_PS_) between individuals using 18 microsatellite loci amplified (a) in 60 modern (b) plus additional seven historical cheetah samples. Modern (solid lines) and historical (dashed lines) samples are colour-coded according their geographical origin. Only bootstrap values (100 reiterations) above 70% are displayed.

In addition to the mitochondrial sequences, we analysed 18 polymorphic microsatellite loci. Using solely modern samples, we assessed the genetic variation among the extant populations according to the clustering solutions with BAPS [mtDNA 915 bp, PP = 1; nuclear DNA (nDNA), PP = 1; Fig. 2b]. The three clusters obtained with mtDNA reflected the geographical distributions of the described subspecies *A. j. jubatus*, *A. j. soemmerringii* and *A. j. venaticus*. At the nuclear level we could define two additional clusters, which represent substructuring within the Southern and Northern-East African subspecies, respectively. We obtained similar clustering results when we visualized the phylogenetic relationship of the individual genotypes in a three-dimensional FCA (Fig. 3). The results from the admixture analysis based on 500 simulations from posterior allele frequencies revealed no admixture (all *P*-values = 1; Fig. 2b) and therefore no evidence of past or present gene flow. The Iranian cheetahs (*H*_E_ = 0.397) were significantly less variable than the Northern-East (*H*_E_ = 0.674) and Southern African (*H*_E_ = 0.698) populations (*P* < 0.001; Wilcoxon rank-sum test corrected for multiple testing). We detected a significant number of loci with heterozygosity excess under the SMM and TPM models, which is consistent with a recent effective population size decline in the Iranian cheetahs. By contrast, no significant signature of a bottleneck was visible in the Southern and Northern-East African populations (Table 5). The population pairwise *F*_ST_/*R*_ST_ values showed significant differentiation between the three populations (*P* < 0.0001; Tables 4 and S3) and the amova results indicated that 22.7% of the total variation occurred among the different populations/subspecies. In a NJ tree (Fig. 4a), the bootstrap support for the branch assembling all modern Asiatic cheetahs was 100% (100 iterations). As all specimen of the East African subspecies *A. j. raineyi* were collected from museum or non-invasively (Table 1), their DNA qualities were not sufficient to retrieve consistent and reliable information over all loci. However, we could obtain nuclear data for seven historical samples (#9, 10, 11, 19, 48, 89, 90; Table 1). When these samples were added to the NJ tree analysis (Fig. 4b) the branch leading to all Southwest Asian samples, which cluster separately from the African individuals (including Libya), had a bootstrap support of 72%.

**Table 5:** Significance of tests for heterozygosity excess assessed using a Wilcoxon sign-rank test under the SMM and TPM model implemented in Bottleneck 1.2.0.2

	*P*-value (SMM)	*P*-value (TPM)
S-West Asia	0.0133	0.0107
N-East Africa	0.8769	0.1733
Southern Africa	0.9700	0.2475

### Estimation of divergence time

We estimated the divergence time, first between Asiatic and Southern African cheetahs, which correspond to the central haplogroup in the mtDNA network, and within Africa, between the best-sampled subspecies *A. j. soemmeringii* and *A. j. jubatus*. Based on the mitochondrial 915-bp fragment, the *D*_A_ (4.412) between *A. j. venaticus* and *A. j. jubatus* was translated into a population split at 41 900 ya (95% CI = 20 300–153 800). Following Gaggiotti & Excoffier (2000), the divergence between these two populations was estimated at 32 170 ya (95% CI = 15 570–118 020). The divergence time between the African subspecies *A. j. soemmeringii* and *A. j. jubatus* was calculated at 66 500 ya (95% CI = 32 200–244 000) and 55 085 ya (95% CI = 26 660–202 100) using *D*_A_ (6.996) and the method of Gaggiotti & Excoffier (2000), respectively. The demographic modelling with IMa suggested a split between *A. j. venaticus* and *A. j. jubatus* at 44 403 ya (90% HPD = 27 420–379 222) and between *A. j. soemmeringii* and *A. j. jubatus* at 72 296 ya (90% HPD = 43 928–379 317). The upper bound for the credibility interval is not informative, as it critically depends on the assumed prior for the maximum value of *t* when the curve slowly decreases to zero after the mode of *t*. The log-likelihood ratio tests did not reject models with *m*1 = *m*2 = 0, which are appropriate for studying the divergence of populations under allopatry (Won *et al.* 2003). Hence, by setting migration rates to zero, we also applied a conventional isolation model (Wakeley & Hey 1997). The splits *A. j.* venaticus/*A. j. jubatus* and *A. j. jubatus*/*A. j. soemmeringii* were then estimated at 42 120 ya (90% HPD = 16 295–83 677) and 66 698 ya (90% HPD = 24 067–117 615), respectively.

Using the microsatellite genetic distance (δμ)^2^ and two human microsatellite mutation rates (2.05 × 10^−3^ and 5.6 × 10^−4^) employed by Driscoll *et al.* (2002), we estimated the split between *A. j. venaticus* and *A. j. jubatus* at 6700 and 24 700 ya, respectively. Using an average mammalian microsatellite mutation rate (2.05 × 10^−4^; Rooney *et al.* 1999), we estimated this divergence at 67 400 ya. The divergence time between *A. j. soemmeringii* and *A. j. jubatus*, using the fastest (human) microsatellite mutation rate, was 3200 ya but rose to 32 400 ya when applying the average mammalian mutation rate. To compare these estimates with another distance method, we used the stepwise-weighted genetic distance *D*_SW_, which is based on the allele frequency differences among populations (Shriver *et al.* 1995). Applying again the two human microsatellite mutation rates and the average mammalian mutation rate, and following Calabrese *et al.* (2001), we calculated the divergence time between Iranian and Southern African cheetahs at 4700, 17 300 and 47 200 ya, respectively. The fastest mutation rate translated into a divergence time estimate among the African subspecies *A. j. soemmeringii* and *A. j. jubatus* of 1600 ya whereas it reached the value of 15 600 ya using the average mammalian mutation rate.

## Discussion

We investigated the genetic diversity and divergence within and between African and Asiatic cheetahs based on mtDNA and microsatellite data of 94 samples including two mediaeval cheetah bones (9th–10th century ce). In general, our data show that there is a higher genetic variation in the current global cheetah population than previously described (O’Brien *et al.* 1983; Menotti-Raymond & O’Brien 1993; O’Brien & Johnson 2005). This is due mainly to the fact that we included populations that had never been investigated before. The overall amount of mtDNA nucleotide diversity (π) of 0.66% in cheetahs was higher than observed in tigers (0.18%; Luo *et al.* 2004) and pumas (0.32%; Culver *et al.* 2000), similar to jaguars (0.77%; Eizirik *et al.* 2001), and lower than in leopards (1.21%; Uphyrkina *et al.* 2001). The total nuclear (microsatellite) diversity (*H*_E_ = 0.766) is comparable with that of other outbred felid species (Culver *et al.* 2000; Eizirik *et al.* 2001; Uphyrkina *et al.* 2001; Driscoll *et al.* 2002). This might be explained by our use of highly polymorphic markers (all loci were polymorphic), therefore we also compared a set of 15 nuclear microsatellite loci applied in surveys of cheetahs (this study and Driscoll *et al.* 2002), domestic cats, pumas and lions (supplemental table 3 in Driscoll *et al.* 2002), and we found similar genetic diversities (*H*_E_ values ranging from 0.681 to 0.777; Table S2) in the four felid species. The *H*_E_ levels in the investigated Southern and Northern-East African cheetah populations (Table 3) were similar to Namibian cheetahs (0.640–0.708) and higher than in the Serengeti population (0.599) (Marker *et al.* 2008). The lower *H*_E_ (0.397) observed in the Iranian cheetahs might be the consequence of ancestral population divergence or a recent bottleneck, as we found significant evidence for a recent effective population size reduction in this population (Table 5).

Within the African samples, we recovered the previously described relationship between the sub-Saharan cheetah subspecies *A. j. raineyi* and *A. j. jubatus* (Menotti-Raymond & O’Brien 1995; Driscoll *et al.* 2002). The clustering of some Tanzanian and Kenyan animals together with Southern African cheetahs in the mtDNA BAPS analysis (Fig. 2a) suggests a population in East Africa that might be derived from relatively recent re-colonization events as observed in lions (Antunes *et al.* 2008). This should be investigated combining mtDNA and nDNA data obtained from additional samples of East African cheetahs. At the nuclear level, we observed substructuring in the Northern-East African and Southern African subspecies (Figs 2b and 3) into two subpopulations, which did not correlate with the geographical origin of the individuals. Weak population structure within the South African cheetahs (Kotze *et al.* 2008) and a panmictic Namibian population (Marker *et al.* 2008) have been reported previously. Remarkably, the Northern-East African cheetahs were highly differentiated (nuclear *F*_ST_ = 0.170) from the Southern African individuals and clustered independently (Figs 1 and 2) and monophyletic (Fig. S1). Between the African and Asiatic subspecies, we also discovered great differentiation at both nuclear and mitochondrial levels (Table 4). Similar levels of population/subspecies differentiation were described in leopards (Uphyrkina *et al.* 2001), pumas (Culver *et al.* 2000) and lions (Antunes *et al.* 2008). We could not detect significantly higher differentiation (mitochondrial *F*_ST_) between African and Asiatic cheetahs than among the African subspecies. This indicates deep phylogeographic structure not only between African and Asiatic cheetahs but also among the African cheetah populations.

It is well documented that imports of tamed hunting cheetahs from Northern-East (Pocock 1939) and East Africa (Divyabhanusinh 2007) into India and the Arabian Peninsula were a regular occurrence during the European colonial era. Given the possibility of interbreeding with African escapees, Asiatic cheetahs were not expected to form a genetically distinct unit. However, hunting cheetahs were highly valued, and there are no known records of individuals (accidentally or intentionally) released into the wild (Divyabhanusinh 2007). Moreover, the species is notoriously difficult to breed in captivity. Except for a single litter born in Akbar’s collection of many thousands of cheetahs the first documented captive birth was at the Philadelphia Zoo in 1956 (Marker & O’Brien 1989; Divyabhanusinh 2007). Therefore, the possibility of a captive, hybrid Asiatic-African population as a source of escapees or releases is very low. In our study, we found no evidence of recent gene flow between these populations (Fig. 2b). In all analyses, including the ones with mediaeval Iranian samples, the Asiatic cheetahs constituted a unique cluster (Figs 1–4) suggesting an apparent monophyly of the Asiatic lineage (Fig. S1). Historical museum specimens from Iran, Iraq and India (#12, 15, 16; Table 1) that could not be distinguished by morphological characteristics from African specimens (Divyabhanusinh 2007) were genetically confirmed as Asiatic individuals.

The clustering results of the Asiatic cheetahs were of particular interest because this population has been proposed to form a single subspecies, *A. j. venaticus*, with North African cheetahs (Ellerman & Morrison-Scott 1951; Krausman & Morales 2005). Notably, Egypt harboured two genetically distinct populations in the past, as we observed clustering of the two Egyptian samples in different haplogroups (Figs 1b and 2a). One now extinct population in eastern Egypt (Northern Sinai; Saleh *et al.* 2001; Hoath 2003) could be represented by the historical sample collected by Theodor von Heuglin in the early 1850s, which clustered with samples from Southwest Asian countries (Jordan, Iran, Iraq and Oman). The other specimen collected in western Egypt (Libyan Desert; Osborn & Helmy 1980) shared the same haplotype with cheetahs from North Africa and represented a population that might still exist today in the Libyan Desert (Saleh *et al.* 2001; Hoath 2003). We could not detect hybridization of Asiatic and African cheetahs in our study (Fig. 2a). This suggests that palaeoclimatic constraints, ecological barriers and/or geographical features prevented past gene flow between the two putative populations of this part of Africa. Genetic separation is also supported by the nuclear NJ tree, as the branch leading to the Jordanian (and all Asiatic) samples, which cluster separately from the Libyan (and all other African) individuals, had a bootstrap support of 72% (Fig. 4b). The classification and taxonomy of North African cheetahs are still debated (Krausman & Morales 2005; Belbachir 2007). This population might be genetically contiguous with cheetahs from West Africa (Senegal to Niger), thus it is critical to further investigate current Egyptian and West African populations as suggested by Belbachir (2007). In the light of our results, the previous proposal of a single subspecies, *A. j. venaticus*, encompassing the Iranian cheetah and its North African congeners (Ellerman & Morrison-Scott 1951; Krausman & Morales 2005) is not supported. In summary, our data based on palaeogenetic analyses demonstrate that the isolation between Asiatic and African cheetahs has existed for millennia.

To date, divergence time between cheetahs has only been estimated among the (closely related; Figs 1 and 2) African subspecies *A. j. jubatus* and *A. j. raineyi*. Using mtDNA (mtRFLP) and microsatellite distance data [(δμ)^2^], the divergence time had been estimated at 28 000–36 000 ya (Menotti-Raymond & O’Brien 1993) and 4253 ya (Driscoll *et al.* 2002), respectively. The latter was inferred with mutation rates estimated from human microsatellite data (Driscoll *et al.* 2002). In general, time estimations based on microsatellite distance data can be challenging, particularly if no taxon-specific microsatellite evolution rates are available, as it is the case in felids. Also, it is important to take into account potential homoplasy of microsatellites (Paetkau *et al.* 1997; Zhivotovsky 2001; Estoup *et al.* 2002) in the estimation of divergence time between ancient isolates, as some cheetah subspecies are suggested to be by our mtDNA divergence time estimates. In this study, we included two newly investigated subspecies (*A. j. soemmeringii* and *A. j. venaticus*) and an average mammalian microsatellite mutation rate previously applied in other felid species (Spong *et al.* 2000; Anderson *et al.* 2004; Ruiz-Garcia *et al.* 2006) to calculate the divergence times within and between African and Asiatic cheetahs. Depending on the genetic marker (mtDNA or nDNA), the nuclear genetic distance [(δμ)^2^ or *D*_SW_] and the choice of the microsatellite mutation rate (human or average mammalian), we retrieved results differing by more than one order of magnitude. Large differences between mitochondrial and nuclear time estimates using human microsatellite rates have been previously observed in wild felids (Menotti-Raymond & O’Brien 1993; Antunes *et al.* 2008). This could be explained by the fact that the genetic distances (δμ)^2^ and *D*_SW_ are considered to underestimate divergence (Paetkau *et al.* 1997; Calabrese *et al.* 2001; Zhivotovsky 2001). In our data, we found higher genetic differentiation at microsatellite loci, as measured by *F*_ST_ values and genetic distances [(δμ)^2^ or *D*_SW_], between *A. j. venaticus* and *A. j. jubatus* than between *A. j. soemmeringii* and *A. j. jubatus*. However, this might be due to a possible stochastic increase in divergence associated with a recent population bottleneck (Chakraborty & Nei 1977; Hedrick 1999), which signature we could apparently detect in our data in *A. j. venaticus*. Choosing the average mammalian mutation rate and considering the mtDNA estimate, we can place the split between Asiatic and African cheetahs at 32 000–67 000 ya and within Africa, between *A. j. soemmeringii* and *A. j. jubatus,* at 16 000–72 000 ya. However, considering the substantial variation in divergence time estimates we acknowledge that decisions for the conservation of this endangered species should not be based on time estimates alone.

### Implications for conservation

In this study, we re-visited the currently recognized cheetah subspecies (Krausman & Morales 2005; Durant *et al.* 2008) in light of our results retrieved from geographically defined populations. We verified the veracity of *A. j. venaticus* and *A. j. soemmeringii* based on recognizable phylogenetic partitioning (mitochondrial monophyly and significant divergence at nuclear loci; Moritz 1994) and absence of gene flow (O’Brien & Mayr 1991). Our study suggests a close relationship of *A. j. raineyi* with *A. j. jubatus*; however, minisatellite (Menotti-Raymond & O’Brien 1993) and microsatellite variation (Driscoll *et al.* 2002) support the separation of these two sub-Saharan subspecies. We also clarified the western range limit of the critically endangered *A. j. venaticus* observing a historical range in contrast to recent accounts, which included North Africa (Krausman & Morales 2005).

The identification of a subspecies recognizes biological distinctiveness and should be sufficient as first-order systematic hypothesis when the aim of conservation is to preserve biological diversity (Green 2005). As large-scale genomic information becomes available also for nonmodel species, adaptive genetic markers might be used to estimate diversification and adaptive genetic variation in subspecies/populations, in combination with supposedly neutrally evolving loci. It might also help to understand how populations can survive despite a low neutral genetic variation (Fraser & Bernatchez 2001; Gebremedhin *et al.* 2009).

Although there is little evidence that inbreeding depression affects African cheetahs (Caro & Laurenson 1994) and current threats to the species are primarily anthropogenic (Nowell & Jackson 1996; Hunter *et al.* 2007; CACP 2008; Marker *et al.* 2008), the lower genetic diversity in the Iranian population is cause for concern in light of their critically low numbers (Hunter *et al.* 2007; Durant *et al.* 2008). Any further decline in Iranian cheetah numbers would require increasingly extreme conservation measures, including the consideration of supplemental introductions from Africa, similar to that required for the demographic rescue of Florida panthers (Creel 2006; Pimm *et al.* 2006; Johnson *et al.* 2010). However, contrary to the close geographic and genetic distances described in Texas and Florida panthers (Johnson *et al.* 2010), we did not detect historical gene flow between African and Asiatic cheetahs (Fig. 2c). In addition to the formidable logistical and financial obstacles arguing against introductions, our results emphasize the importance of preserving the genetic distinctiveness of the critically endangered Iranian cheetahs. That will also entail a stronger understanding of possible substructuring in the Iranian population. At least 10 cheetahs moving between known population centres have been killed on roads since 2004 including most recently a female with two cubs in August 2010 (A. Jourabchian, unpublished data). Ongoing, rapid infrastructural development around some cheetah subpopulations in Iran will increase the likelihood of demographic and genetic fragmentation. These processes are currently poorly understood but are the focus of an ongoing multinational research effort led by Iranian biologists which has deployed GPS collars on cheetahs and is undertaking further analysis of genetic differences between cheetah subpopulations in Iran (Hunter *et al.* 2007). Most significantly, the government of Iran recently renewed its commitment to a major conservation effort of the species (Breitenmoser *et al.* 2010).

Our results have particular implications for proposed reintroductions in the cheetah’s former range in Asia, especially in India (Ranjitsinh & Jhala 2010). Such endeavours face massive challenges of habitat and prey availability but assuming these are overcome, the question of the founder’s origin remains. The genetic distinctiveness of Asiatic cheetahs would argue that reintroduction efforts should attempt to use cheetahs from Iran. However, this population is critically endangered and cannot sustain removals. Both Southern and Northern-East African cheetahs would have sufficient genetic variability to be considered as independent source populations. Thus, the choice of the most promising source population should be based on ecological, behavioural and viability criteria with minimum taxonomic swamping. In any case, the current Iranian cheetahs will probably remain the only representatives of the subspecies *A. j. venaticus* in Asia for the foreseeable future.

## Conclusion

Current conservation and management strategies are usually based on the recognition of the subspecies taxonomy. In the case of the cheetah this has rarely been considered, as cheetahs were found to have little genetic variation (Menotti-Raymond & O’Brien 1995; O’Brien & Johnson 2005). Our data suggest this viewpoint to be valid for the two sub-Saharan subspecies *A. j. jubatus* and *A. j. raineyi*, which could not be entirely separated in the mitochondrial population structure analysis. However, we show that Northern-East African, Southern African and Asiatic cheetahs are long-term geographic isolates with independent evolutionary histories. Moreover, we demonstrate that the critically endangered Iranian cheetahs are an autochthonous, monophyletic population and the last representatives of the Asiatic cheetah. Our data also support the view of an independent subspecies status for the cheetahs in North Africa. This population may be genetically contiguous with those from West Africa (Senegal–Niger), historically classified as *A. j. hecki*, but additional sampling is required to resolve this issue (Belbachir 2007). Also, it will be important to survey adaptive genetic variation in the cheetah subspecies to better understand evolutionary differentiation caused by ecological adaptation. Based on our results, we conclude that unique diversity remains in the cheetahs of Africa and Asia and that conservation of these populations, especially of the critically low numbering Iranian individuals, should rank high among felid conservation priorities.

## Supporting information

**Table S1** Mitochondrial fragments amplified for aDNA analysis **Table S2** Comparisons of average numbers of alleles per locus and expected heterozygosities calculated from 15 microsatellite loci overlapping in four different felid species. **Table S3** Population pairwise distances (*R*_ST_) based on 18 polymorphic microsatellite loci. **Fig. S1** Phylogenetic relationship based on a ML tree inferred from the 915-bp concatenated mtDNA fragment.
