# Supplementary material for: Phylogeography, genetic structure and population divergence time of cheetahs in Africa and Asia: evidence for long-term geographic isolates
Source: Mol Ecol. 2011 Feb;20(4):706–24. doi: 10.1111/j.1365-294X.2010.04986.x (PMC3531615; doi:10.1111/j.1365-294X.2010.04986.x)
Supplement: Supplementary file 1 — Table S1 Mitochondrial fragments amplified for aDNA analysis Table S2 Comparisons of average numbers of alleles per locus and expected heterozygosities calculated from 15 microsatellite loci overlapping in four different felid species. Table S3 Population pairwise distances (RST) based on 18 polymorphic microsatellite loci. Fig. S1 Phylogenetic relationship based on a ML tree inferred from the 915-bp concatenated mtDNA fragment. [file mec0020-0706-SD1.doc]

Supporting Information

Core Facility of Palaeogenetics, Paris

The ancient DNA (aDNA) analysis of the medieval cheetah bone specimens was carried out in the Core Facility of Palaeogenetics at the Jacques Monod Institute (IJM), Paris. This highly contained and pressurized laboratory dedicated to aDNA analysis is isolated on the sixth floor of the institute where no other molecular biology laboratories are located. It consists of an airlock chamber and three different laboratory rooms dedicated to different specific steps of the experimental procedures, (i) bone preparation, (ii) DNA extraction, (iii) PCR set-up. The rooms are subjected to a positive air pressure gradient. Every separate working station and items are cleaned with bleach (3.5% hypochlorite solution) or RNase away® (Molecular Bio-Products, USA) and UV irradiated between each experiment. To minimize contamination with exogenous DNA and maximize aDNA yield, we used, for extraction and PCR preparation, low retention microtubes (Axygen, Union City, USA) and extra-long pipette tips.

Experimenters only enter the laboratory after having removed their street clothes and replaced them with bleach-washed clothes. During each step of pre-PCR work an all-body protection (a different one for each laboratory room) consisting of over-all protection clothing, two pairs of gloves, cover shoes and facemask, is worn. Purification was carried out in a flow hood, and pipetting for the PCRs in a template tamer that were UV irradiated after each experiment. Capillaries containing the reagent mixtures for the PCR and the fossil extracts were closed in the template tamer of the fourth room of the contained laboratory prior to transfer to the PCR machines in a laboratory on the 5th floor of the building. The handling of post-PCR products was exclusively done on the 5th floor of the building in a separate and dedicated laboratory.

Quantitative Real-Time PCR of aDNA

Ancient DNA was amplified by UNG-coupled quantitative real-time PCR (UQPCR) using the LightCycler® FastStart DNA MasterPLUS SYBR Green I mix (Roche Diagnostics GmbH, Roche Applied Science, Mannheim, Germany). Briefly, to minimize carry-over contamination, dTTP was replaced by dUTP in the PCR mix and a digestion with uracil-N-glycosylase (UNG) was performed prior to each PCR to cleave any contaminating PCR products originating from previous PCR steps. At the same time, this step also cleaves the main diagenetic lesions, deaminated cytosine, thus increasing the reliability of the obtained sequences. Amplifications were carried out in individual glass capillaries. Quantification of initial target molecules was performed for each PCR using a titration curve established with a homologous reference (DNA from a Namibian lineage specimen, *A. j. jubatus*) according to Pruvost et al. (2005, 2007). Inhibition of the polymerase by the extracts was evaluated and quantified for each extract following Pruvost and Geigl (2004), but using as homologous internal standard *A. j. jubatus* DNA as previously described. For the evaluation of the inhibition three parameters were taken into consideration: (i) delay of the threshold value Ct (crossing point at threshold), (ii) the kinetics of the synthesis of the PCR product, and (iii) the efficiency of the PCR. The quantity of aDNA extract amplified by PCR was adjusted according to the results of the inhibition tests to minimize interference of the inhibitors with the PCR. Identification and characterization of the PCR products were performed by analysis of the fusion temperature (Tm) of the products using the Lightcycler® and via electrophoresis of 1 µL of the qPCR product in a 10% polyacrylamide gel. PCR products of the expected Tm and size in the gel were purified using the QIAquick PCR purification kit (Qiagen) and sequenced by Eurofins MWG GmbH (Ebersberg, Germany).

Authentication of aDNA sequences

For each ancient sample two independent extractions were performed separated in time. Each extract was amplified with several primers and each PCR product was obtained at least twice. We sequenced 139 bp including 14 informative polymorphisms in MT-ND5 and MT-CR (Table 2). All sequences were compared to the reference mitogenome of *Acinonyx jubatus* [Accession number GI:38349475.1]. The same sequences were obtained from the two different bones (mandible and vertebra) from Tahkt-e Suleyman indicating that they belonged to one individual, a result that is not contradicting the palaeontological determination.

# Table S1. Mitochondrial fragments amplified for aDNA analysis

| Amplicons | Length (bp) | Position 5' | Position 3' |
| --- | --- | --- | --- |
| aMT-ND5 | 53 | 12657 | 12709 |
| aMT-CR1 | 41 | 16444 | 16484 |
| aMT-CR3 | 45 | 16812 | 16856 |
| Total | 139 |  |  |

Numbering according to Genbank [Accession number GI:38349475.1].

**Table S2.** Comparisons of average numbers of alleles per locus and expected heterozygosities calculated from 15 microsatellite loci* overlapping in four different felid species.

| *15 overlapping loci* | | Average number Allele/Locus (SE) | H_e_ |
| --- | --- | --- | --- |
| **Cheetahs** |  |  |  |
| Driscoll *et al.* (2002) | |  |  |
|  | *Acinonyx jubatus* (all) | 7.067 (1.534) | 0.758 |
|  | *A.j.jubatus* (n=20) | 5.533 (1.060) | 0.711 |
|  | *A.j.raineyi* (n=10) | 5.067 (1.223) | 0.678 |
|  |  |  |  |
| Marker *et al.* (2008)** | |  |  |
|  | *A.j.jubatus* (n=89) | 6.857 (1.351) | n.a. |
|  |  |  |  |
| current study |  |  |  |
|  | *Acinonyx jubatus* (all) | 8.267 (1.438) | 0.777 |
|  | *A.j.jubatus* (n=27) | 6.467 (1.598) | 0.687 |
|  | *A.j.soemmeringii* (n=25) | 5.933 (1.486) | 0.692 |
|  | *A.j.venaticus* (n=8) | 2.333 (1.047) | 0.402 |
|  |  |  |  |
| **Domestic cats** (Driscoll *et al.*; 2002) | |  |  |
|  | *Felis catus* (n=10) | 5.867 (2.031) | 0.704 |
|  |  |  |  |
| **Lions** (Driscoll *et al.*; 2002) | |  |  |
|  | *Panthera leo* (n=60) | 6.467 (2.356) | 0.681 |
|  |  |  |  |
| **Pumas** (Driscoll *et al.*; 2002) | |  |  |
|  | *Puma concolor* (n=30) | 7.733 (1.751) | 0.695 |

* 15 loci: FCA005, FCA008, FCA014, FCA026, FCA069, FCA085, FCA097, FCA105, FCA126, FCA133, FCA212, FCA224, FCA230, FCA247, FCA310

**14 loci (without FCA005)

**Table S3.** Population pairwise distances *R_ST_*  based on 18 polymorphic microsatellite loci.

|  | N-East Africa (n=25) | Southern Africa (n=27) |
| --- | --- | --- |
| S-West Asia (n=8) | 0.216 | 0.455 |
| N-East Africa | _- | 0.226 |

*R_ST_* were calculated in Arlequin 3.5. *R_ST_* p-values are significant (p < 0.0001). Populations were defined according BAPS and FCA clustering.

**Figure S1. Phylogenetic relationship based on a ML tree inferred from the 915-bp concatenated mtDNA fragment.** The branches of the ML consensus tree with bootstrap support lower than 50% are collapsed.


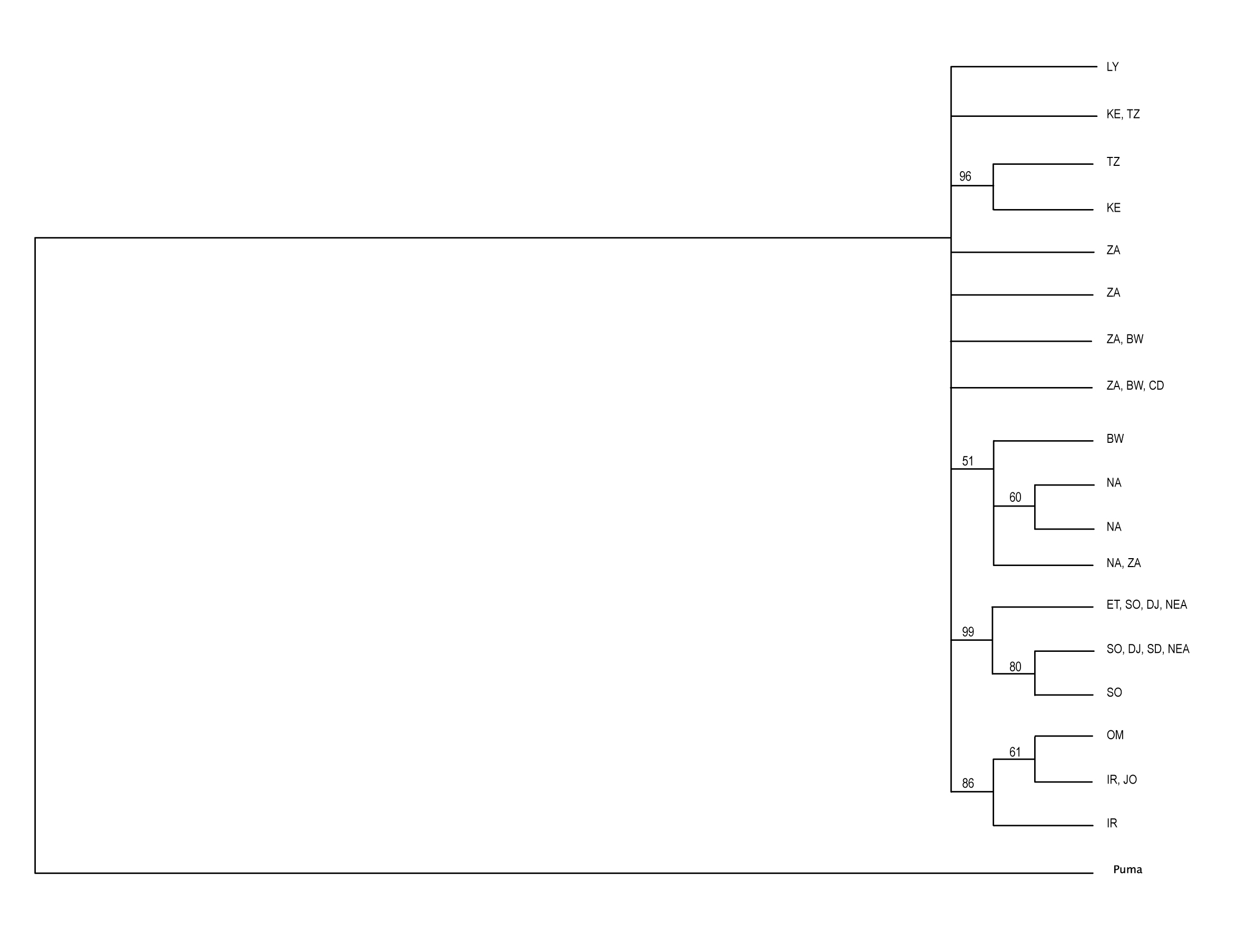
Names of the haplotypes are coded following ISO 3166-Alpha 2.

**N.E-AFRICA**

**S.W-ASIA**
